# Validation of the Pediatric Advanced Weight Prediction in the Emergency Room Extra Large Mid-Arm Circumference scale in Turkish children

**DOI:** 10.3389/fped.2026.1915860

**Published:** 2026-09-16

**Authors:** Yesim Andiran Senayli, Sevgi Ulusoy Tangul, Akif Demirel, Atilla Senayli

**Affiliations:** 1 School of Medicine, Department of Anesthesiology and Reanimation, Yozgat Bozok University, Yozgat, Türkiye; 2 School of Medicine, Department of Pediatric Surgery, Yozgat Bozok University, Yozgat, Türkiye

**Keywords:** habitus score, mid-arm circumference, PAWPER XL-MAC, Turkish children, validation, weight estimation

## Abstract

**Aim:**

This study aimed to validate the Pediatric Advanced Weight Prediction in the Emergency Room Extra Large-Mid-Arm Circumference (PAWPER XL-MAC) weight estimation scale in Turkish children by evaluating its accuracy, agreement, and validity across age groups. We assessed the scale's performance using mid-arm circumference (MAC), habitus status (HS), and height to predict weight, while examining age- and gender-related differences.

**Methods:**

This study included pediatric patients (aged 0–18 years) who presented to a pediatric surgery outpatient clinic from December 2024 to April 2025. Data on demographics, height, weight, MAC, and clinician-assigned HS (1–7) were collected. PAWPER XL-MAC estimated weights were calculated. Accuracy was assessed using percentage within 10% (PW10) and percentage within 20% (PW20). Analyses included descriptive stats, Pearson correlations, *t*-tests/Welch's *t*-tests, ANOVA, multiple regression, Bland–Altman plots, and confirmatory factor analysis (CFA) for validity measures. SPSS, R, and Jamovi were used.

**Results:**

The cohort comprised 323 boys (65.8%) and 168 girls (34.2%), for a total of 491 patients, with infants (0–2 years) forming the largest group (41.8%). Significant gender differences were observed in MAC and weight (*p* < 0.01). The multiple linear regression model (weight∼MAC + height + HS) demonstrated a strong fit (R^2^ = 0.878). Overall, PW10 was 10.8%, and PW20 was 21.0%, with marked age-dependent variation, with moderate performance in schoolers (PW10: 10.1%, PW20: 22.2%) and the highest in adolescents (PW10: 41.9%, PW20: 81.7%). MAC–HS agreement showed a moderate-to-weak positive association (gamma = 0.226). PCA revealed a single dominant factor explaining 62.4% of variance, with strong loadings for weight (0.89) and MAC (0.85). CFA proxy indices indicated acceptable-to-good model fit (comparative fit index ∼0.92–0.95, standardized root mean square residual ∼0.06–0.08).

**Conclusion:**

The PAWPER XL-MAC scale shows acceptable structure validity in Turkish children but has age-related accuracy issues, especially in younger groups. Age-specific adjustments are recommended to improve reliability for emergency weight estimation and drug dosing in children.

## Introduction

Accurate body weight estimation in pediatric emergency care is critical for medication dosing, fluid therapy, and resuscitation interventions (1–5). Traditional age-based formulas or length-based tapes have well-documented limitations, particularly in contemporary populations where obesity and malnutrition rates are rising (5). In response, more advanced tools that incorporate body habitus assessment have been developed (1, 3, 5). The most recent of these is the integration of mid-arm circumference (MAC) measurement into weight prediction systems (3, 5, 6).

The Pediatric Advanced Weight Prediction in the Emergency Room Extra Length/Extra Large Mid-Arm Circumference (PAWPER XL-MAC) scale combines height, a clinician-assigned habitus status (HS 1–7), and an objective MAC measurement to provide personalized weight estimation (2, 7). The scale allows dual assessment of habitus through visual inspection and MAC-based cutoff values, aiming to improve accuracy, especially in underweight and obese children (2, 8).

International studies have generally reported high accuracy for the PAWPER XL-MAC scale, with estimates within 10% of actual weight in more than 78% of cases and within 20% in 96% of cases in high-income country settings (1, 6, 9). However, performance can vary across populations due to differences in body composition, nutritional status, and obesity prevalence (4, 5, 10). In Turkey, where childhood obesity and nutritional problems show distinct regional patterns, the validity and performance characteristics of the PAWPER XL-MAC scale remain unknown.

This prospective observational study aimed to validate the PAWPER XL-MAC scale in Turkish pediatric patients aged 0–18 years. The study evaluated agreement between visually assigned habitus scores and MAC-derived categories; assessed weight-estimation accuracy using the percentage within 10% (PW10) and percentage within 20% (PW20) metrics across four age groups (infants, preschool, school-age, and adolescents); and examined structural validity using multiple linear regression, Bland–Altman analysis, and single-factor principal component analysis (PCA) with confirmatory factor analysis (CFA) proxy metrics. Demographic characteristics, gender differences, and correlations among age, MAC, habitus score, and actual weight were also analyzed.

Our hypothesis was that MAC would show a strong positive relationship with the habitus score, that weight ratio (actual/ideal weight) would vary in parallel with habitus categories, and that the key variables (habitus status, MAC, weight, and weight ratio) would cluster under a single latent “body habitus” factor, thereby supporting the structural validity of the scale in the Turkish pediatric population.

## Method

### Study design and participants

The study was conducted by examining the data of patients who visited a pediatric surgery outpatient clinic between December 2024 and April 2025. Demographic information (age, gender), height, weight, mid-arm circumference, and habitus score measurements of the patients were collected. Pediatric patients aged 0−18 years were included.

### PAWPER XL-MAC application and anthropometric measurements

The measured length/height and the assigned HS were used to obtain the estimated weight on the PAWPER XL-MAC tape as defined in the original guideline. HS was then assigned by a clinician via visual assessment on a 7-point scale from 1 (very thin/cachectic) to 7 (extremely obese), using the predefined visual descriptors of the PAWPER XL-MAC system. MAC was measured at the midpoint between the acromion and olecranon processes of the non-dominant arm using a non-stretchable measuring tape. After the estimation procedure was completed, actual body weight was measured using a calibrated digital pediatric scale (Seca 376 or equivalent calibrated platform scale commonly used in Turkish pediatric clinics), with the child standing barefoot and wearing only light clothing or, for infants, lying in a tared infant weighing tray. All measurements were performed by trained clinicians following a standardized protocol to minimize inter-observer variability.

HS assessment was performed visually by the clinician, scoring between 1 (very thin/cachectic) and 7 (extremely obese) according to the PAWPER XL-MAC guideline. The HS percentile equivalents are as defined in the guideline as follows: HS 1 (<5th percentile), HS 2 (5−15th percentile), HS 3 (∼50th percentile, ideal), HS 4 (85−95th percentile), and HS 5−7 (>95th percentile). PAWPER XL-MAC estimated weight was calculated using height, MAC measurement, and HS.

Continuous variables (weight, MAC, and HS) are expressed as means, medians, standard deviations, and minimum–maximum ranges. Categorical variables, including gender and age groups, are presented as frequencies and percentages. The study sample was categorized into the following four distinct age groups: 0–2 years (infant), 2–6 years (preschool), 7–12 years (school-age), and 12 years or older (adolescent).

Weight ratio was calculated as the actual measured body weight divided by the ideal body weight (determined via quadratic interpolation based on height and the 50th percentile reference values according to the PAWPER XL-MAC guideline). Weight estimation accuracy was evaluated using PW10 and PW20 of the actual weight. These metrics are widely used, with common thresholds for acceptable performance being PW10 > 70% and PW20 > 95% (1). Higher values indicate better performance. Based on pediatric emergency validation frameworks, global accuracy rates of PW10 > 70% and PW20 > 95% are recognized as the standard clinical target for an “acceptable” performance. Subgroup analyses by age were qualitatively categorized from “lowest performance” to “highest performance” based on their relative proximity to these established international accuracy benchmarks, reflecting the known challenges of weight estimation in younger pediatric cohorts.

### Statistical analysis

Data were analyzed using SPSS, R 4.6.0, and the Jamovi 2.7.32 package.

Descriptive statistics for normally distributed continuous variables are presented as mean ± standard deviation (SD), while categorical variables are presented as frequencies and percentages. Demographic data, including sex, were recorded for all participants. Although the primary analyses were performed on the pooled cohort, supplementary sex-stratified analyses were conducted to evaluate potential variability in performance between boys and girls. The normality of the data and homogeneity of variances were assessed using the Shapiro–Wilk and Levene's tests, respectively. To investigate whether measurements differed by gender, independent-samples *t*-tests were conducted. Due to a violation of the assumption of equal variances for weight, Welch's *t*-test was utilized for parametric comparisons, while the Mann–Whitney U test was also used as a non-parametric alternative.

To examine the relationship between age (as a continuous variable) and the body measurements (HS, MAC, and weight), Pearson correlation analysis was performed. Statistical significance was set at *p* < 0.05.

Intergroup comparisons were planned with Student’s *t*-test or one-way ANOVA when appropriate; however, the primary focus of this validation study was on agreement and structural validity metrics rather than direct hypothesis testing between multiple groups.

A multiple linear regression model was constructed to predict actual body weight using MAC, height, and HS as predictors (Weight∼MAC + height + HS). Model fit was evaluated using the coefficient of determination (R^2^) and adjusted R^2^. Parameter estimates, standard errors, *t*-statistics, and *p*-values are reported for each predictor. Multicollinearity was assessed using variance inflation factors (VIFs), with values <5 considered ideal and <10 acceptable.

Model assumptions were verified as follows: normality of residuals via Shapiro–Wilk, Kolmogorov–Smirnov, and Anderson–Darling tests, supplemented by Q-Q plots; homoscedasticity via Breusch–Pagan and Goldfeld–Quandt tests; and independence of observations via the Durbin–Watson statistic.

### Structural validity assessment

Agreement metrics were defined as follows: MAC-HS agreement rate refers to the percentage of cases in which the visually assigned HS by the clinician matched the HS category derived from the measured MAC according to the pre-defined cut-off values in the PAWPER XL-MAC guideline. Weight estimation agreement was assessed by comparing the PAWPER XL-MAC-estimated weight with the actual measured weight using several complementary approaches: mean bias, Bland–Altman plots with 95% limits of agreement, and PW10 and PW20 of actual weight. Single-factor PCA and CFA proxy metrics, including the comparative fit index (CFI), Tucker–Lewis Index (TLI), root mean square error of approximation (RMSEA), and standardized root mean square residual (SRMR), were employed to evaluate the structural (construct) validity of the PAWPER XL-MAC scale in the Turkish pediatric population. In the context of scale validation, PCA assesses the underlying dimensionality and variance explained by a single dominant factor, while the CFA proxy indices provide complementary information on model fit. Conventionally, CFI and TLI values greater than 0.90 (preferably >0.95) indicate good fit, RMSEA values below 0.08 (ideal < 0.06) suggest acceptable approximation error, and SRMR values under 0.08 reflect minimal residual discrepancies.

These metrics collectively demonstrate the extent to which the PAWPER XL-MAC scale’s components (habitus score and mid-arm circumference) align with a unified latent construct of weight estimation accuracy, thereby supporting or challenging its structural validity for clinical use in this demographic.

### Ethics approval

This prospective observational study was approved by the Yozgat Bozok University Non-Interventional Clinical Research Ethics Committee (GOKAEK) with decision number 183 dated 06.11.2024. All eligible patients or their parents were contacted by their doctor to object to or consent to the use of their data. All parents of the children enrolled in this study gave consent to participate. This study adhered to the Declaration of Helsinki.

## Results

### Demographic characteristics of the study population

A total of 491 patients with complete gender data were included in the final descriptive analysis. The overall study population consisted of 323 boys (65.8%) and 168 girls (34.2%). Regarding the age distribution, the largest subset was the infant group (0–2 years) with 205 patients (41.8%), followed by school-age children (7–12 years; *n* = 99, 20.2%), preschool children (2–6 years; *n* = 94, 19.1%), and adolescents (aged 12 years or older; *n* = 93, 18.9%) (Table 1).

**Table 1:** Anthropometric characteristics (habitus score, mid-arm circumference, and body weight) according to age group among Turkish pediatric patients (*N* = 491).

Age group	Variable	*N*	Mean	Median	Standard deviation (SD)	Minimum (Min.)	Maximum (Max.)
Infant (0–2 years)	HS MAC Weight	205 205 205	2.65 15.0 10.1	3 15 9.00	0.674 2.25 7.17	1 8 3.00	4 20 90.0
Preschool (2–6 years)	HS MAC Weight	94 94 94	2.36 16.9 18.3	2.00 16.0 18.0	0.686 2.31 5.49	1 12 7.00	5 22 37.0
School-age (7–12 years)	HS MAC Weight	99 99 99	2.49 19.9 33.2	2 19 31.0	0.774 3.38 10.5	1 12 19.0	5 28 68.0
Adolescent (12+ years)	HS MAC Weight	93 93 93	2.96 25.4 60.1	3 25 57.0	1.08 4.39 16.9	1 17 27.0	7 39 109

HS, habitus score; MAC, mid-arm circumference.

### Anthropometric measurements and gender comparisons

Pearson's chi-square test demonstrated a statistically significant association (*p* < 0.01) between gender distribution and age categories. There were predominantly more boys in the infant (73.2% male vs. 26.8% female) and preschool (74.5% male vs. 25.5% female) groups. Conversely, a relatively balanced distribution with a slight female predominance was observed in the adolescent group (53.8% female vs. 46.2% male) (Table 2).

**Table 2:** Gender distribution across age groups in Turkish pediatric patients (*N* = 491).

Age group	Gender	
	M	F
Infant (0–2 years)		
Observed	150	55
% within column	46.4%	32.7%
Preschool (2–6 years)		
Observed	70	24
% within column	21.7%	14.3%
School-age (7–12 years)		
Observed	60	39
% within column	18.6%	23.2%
Adolescent (12 years or older)		
Observed	43	50
% within column	13.3%	29.8%
Total		
Observed	323	168
% within column	100.0%	100.0%

*Within-column* % = (cell count/column total) × 100. Pearson's chi-square test was used to evaluate associations between variables. Variable associations were evaluated via Pearson's chi-square test (*p* < 0.01). M, male; F, female.

Comparisons of body measurements based on gender were evaluated (Table 3). The analysis revealed no statistically significant difference between male and female children regarding HS according to Welch's *t*-test [*t* (354) = −0.450, *p* = 0.653]. Conversely, a statistically significant difference was observed in MAC [*t* (318) = −2.940, *p* = 0.004]. Similarly, weight measurements demonstrated a highly significant difference between genders [*t* (311) = −3.636, *p* < 0.001], indicating that gender plays a critical role in weight and MAC variation (Figure 1).

**Table 3:** Comparison of habitus status, mid-arm circumference, and body weight between male and female Turkish pediatric patients.

Variable	Statistic	df	*p*
HS			
Student's *t*	−0.442	488	0.658
Welch's *t*	−0.450	354	0.653
Mann–Whitney U	26,032		0.453
MAC			
Student's *t*	−3.006	488	0.003
Welch's *t*	−2.940	318	0.004
Mann–Whitney U	22,297		0.001
Weight			
Student's *t*	−3.749^a^	488	<0.001
Welch's *t*	−3.636	311	<0.001
Mann–Whitney U	21,353		<0.001

Data were analyzed using Student's *t*, Welch's *t*, and Mann–Whitney U tests based on the normality of the distribution.

a The adjusted statistical value according to Levene's test since the assumption of homogeneity of variances was not met. Statistical significance was set at *p* < 0.05.

HS, habitus score; MAC, mid-arm circumference; df, degrees of freedom.

**Figure 1:**
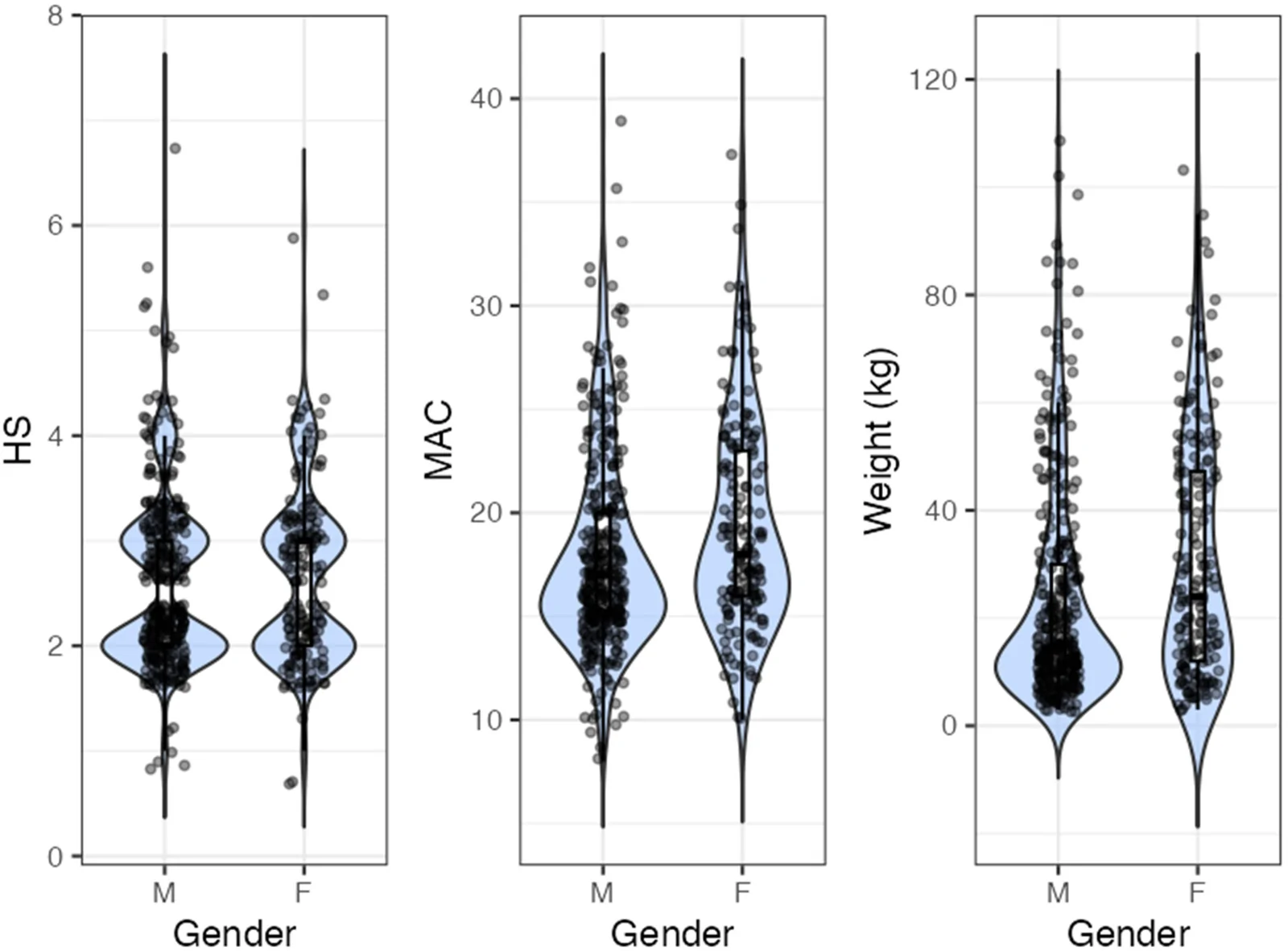
Violin plots showing the distribution and comparison of HS, MAC, and weight measurements by gender.

### Habitus score groups and MAC–HS agreement

Pairwise comparisons using the Games–Howell *post-hoc* test indicated that height significantly differed in the HS 5 group, where patients had higher mean height values compared to those in the HS 2 (*p* = 0.018), HS 3 (*p* = 0.008), and HS 4 groups (*p* = 0.035). Regarding body weight, the mean values in HS 5 were significantly higher than those in HS 1 (*p* = 0.020), HS 2 (*p* = 0.028), and HS 3 (*p* = 0.036). Additionally, patients in the HS 4 group demonstrated significantly greater body weight than those in the HS 2 group (*p* = 0.013). Concerning MAC values, the *post-hoc* analysis highlighted distinct clustering. Both the HS 4 and HS 5 groups exhibited significantly higher mean MAC values than the lower habitus strata, namely the HS 1, HS 2, and HS 3 groups (*p* < 0.05 for all respective dual comparisons). No statistically significant differences were observed among the HS 1, HS 2, and HS 3 group pairs across any of the analyzed variables (*p* > 0.05) (Table 4). Due to insufficient sample sizes yielding an inadequate number of observations for variance computation, the patients categorized into the HS 6 (*n* = 2) and HS 7 (*n* = 1) groups were excluded from the comparative inference testing and were only evaluated descriptively.

**Table 4:** Comparison of height, body weight, and mid-arm circumference across habitus status categories in Turkish children.

Variable	HS 1 *(n* = *9)*	HS 2 *(n* = *243)*	HS 3 *(n* = *178)*	HS 4 *(n* = *51)*	HS 5 *(n* = *7)*	*p*-value	Post-hoc pairwise comparisons (Games-Howell, *p* < 0.05)
Height (cm) Mean ± SD	98.4 ± 41.3	118.2 ± 14.7	135.4 ± 21.8	142.1 ± 14.3	148.3 ± 29.1	<0.001	HS 5 > HS 2, HS 3, HS 4
Weight (kg) Mean ± SD	12.8 ± 14.4	18.5 ± 14.7	26.8 ± 21.8	33.5 ± 28.7	45.2 ± 29.2	<0.001	HS 5 > HS 1, HS 2, HS 3; HS 4 > HS 2
MAC (cm) Mean ± SD	15.67 ± 14.4	17.32 ± 1.5	18.09 ± 2.8	21.78 ± 2.9	29.71 ± 2.8	<0.001	HS 4 > HS 1, HS 2, HS 3; HS 5 > HS 1, HS 2, HS 3

SD, standard deviation; MAC, mid-upper-arm circumference; HS, habitus score. Statistical analyses were performed using one-way ANOVA (Welch's specification) followed by the Games–Howell *post-hoc* test. Due to insufficient sample sizes that prevented variance estimation, participants classified in the HS 6 (*n* = 2) and HS 7 (*n* = 1) groups were removed from the inferential comparative analysis.

As shown in the table, height, weight, and MAC differed significantly across Habitus Score groups (*p* < 0.001 for all parameters), indicating that physical measurements systematically increased with higher habitus scores.

Although the MAC–HS agreement rate was high in the HS 1 group (88.9%), this finding should be interpreted cautiously due to the small sample size in this category (*n* = 9). Similarly, the extremely obese categories (HS 5–7) were represented by only 10 patients in total.

The anthropometric characteristics and MAC values across different HS groups are detailed in Table 4. As stated above, due to insufficient sample sizes yielding an inadequate number of observations for variance computation, the patients categorized into the HS 6 (*n* = 2) and HS 7 (*n* = 1) groups were excluded from the comparative inference testing and were only evaluated descriptively. One-way Welch's ANOVA revealed highly significant overall differences among the remaining five HS groups regarding height, weight, and MAC measurements (*p* < 0.001).

To evaluate the global ordinal diagnostic agreement across the entire spectrum of the pediatric sample, a cross-tabulation analysis incorporating all seven HS tiers was performed. Utilizing Goodman–Kruskal's gamma to assess the correlation between the MAC stratification method and the reference HS, the test yielded a stable coefficient value of 0.226. Based on standard ordinal association indices, this outcome indicates a statistically significant, moderate-to-weak positive association. The finding implies that while increasing MAC measurements successfully parallel higher somatic classifications globally, precise diagnostic boundaries occasionally overlap at the individual level (Table 5).

**Table 5:** Ordinal agreement between mid-arm circumference-derived categories and clinician-assigned habitus scores using Goodman–Kruskal’s gamma in Turkish pediatric patients.

Analysis component	Test metric	Value	Interpretation
MAC vs. full reference HS (Tiers 1–7)	Goodman–Kruskal’s gamma	0.226	Moderate-to-weak positive association

MAC, mid-upper-arm circumference; HS, habitus score. Unlike the parametric variance testing, this ordinal alignment model incorporates the complete sample across all seven habitus strata.

### Correlations and multiple linear regression analysis

Pearson correlation analysis indicated a statistically significant but weak positive relationship between age and HS (*r* = 0.107, *p* = 0.018) (Table 6). In contrast, age exhibited a very strong and statistically significant positive correlation with MAC (*r* = 0.808, *p* < 0.001) and an exceptionally strong positive correlation with weight (*r* = 0.875, *p* < 0.001). These results demonstrate that while habitus scores change minimally with age, weight and mid-arm circumference increase substantially as children grow older. Scatter plot matrices show the linear regression trends (Figure 2). The multiple linear regression model (Weight∼MAC + height + HS) demonstrated strong explanatory power (R^2^ = 0.878, *p* < 0.001). Multicollinearity diagnostics showed acceptable VIF values (MAC: 2.84; height: 3.12; HS: 1.67), indicating no serious multicollinearity issues despite the known relationship between HS, MAC, and height. Residual diagnostics confirmed that the assumptions of linearity, homoscedasticity, and normality were reasonably satisfied. Multicollinearity diagnostics revealed acceptable VIF values overall (HS: 1.58; height: 4.38), though MAC was slightly elevated (VIF = 5.17). As all values remained well below the conventional threshold of 10, multicollinearity was not considered to critically affect model stability.

**Table 6:** Pearson correlation matrix of age, habitus score, mid-arm circumference, and body weight.

Variable		HS	MAC	Age
MAC	Pearson's *r*	0.399***	–	–
Age	Pearson's *r*	0.107*	0.808***	–
Weight	Pearson's *r*	0.317***	0.901***	0.875***

*p < 0.05.

***p < 0.001.

**Figure 2:**
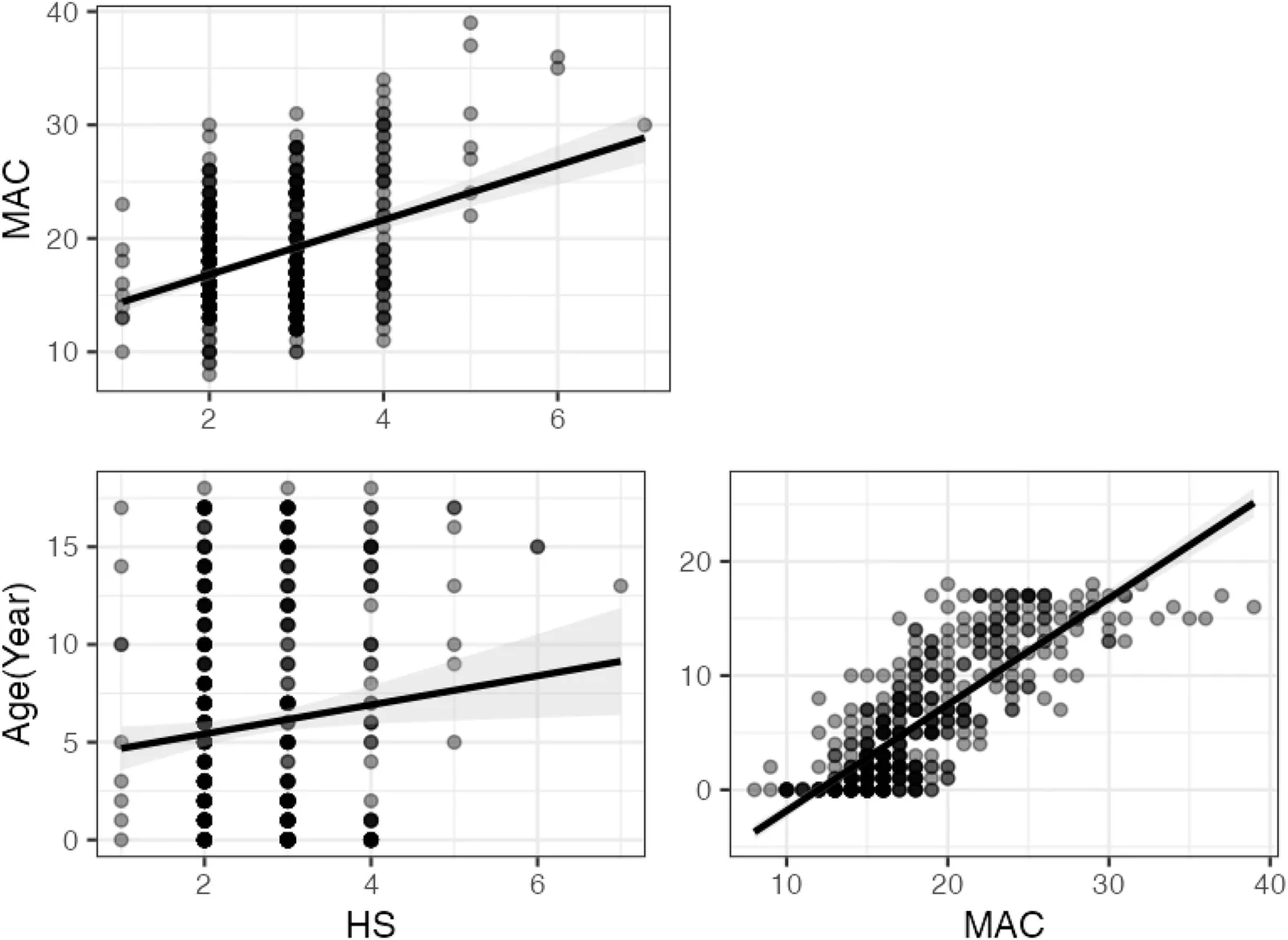
Scatter plot matrices illustrating the linear regression trends and correlations between age and developmental parameters (HS and MAC).

Residual diagnostics confirmed that model assumptions were largely satisfied. Although normality tests indicated significant deviations (Shapiro–Wilk *p* < 0.001; Kolmogorov–Smirnov *p* < 0.001; Anderson–Darling *p* < 0.001), visual inspection of the Q–Q plot showed that the majority of residuals followed the diagonal reference line closely, with deviations limited to the tails and attributable to a small number of outliers (Figure 3). Given the large sample size, the Central Limit Theorem supported the robustness of the regression estimates. Homoscedasticity was not violated in the Breusch–Pagan test (statistic = 6.22, *p* = 0.102). The Durbin–Watson statistic (1.98, *p* = 0.816) indicated no significant autocorrelation (Table 7).

**Figure 3:**
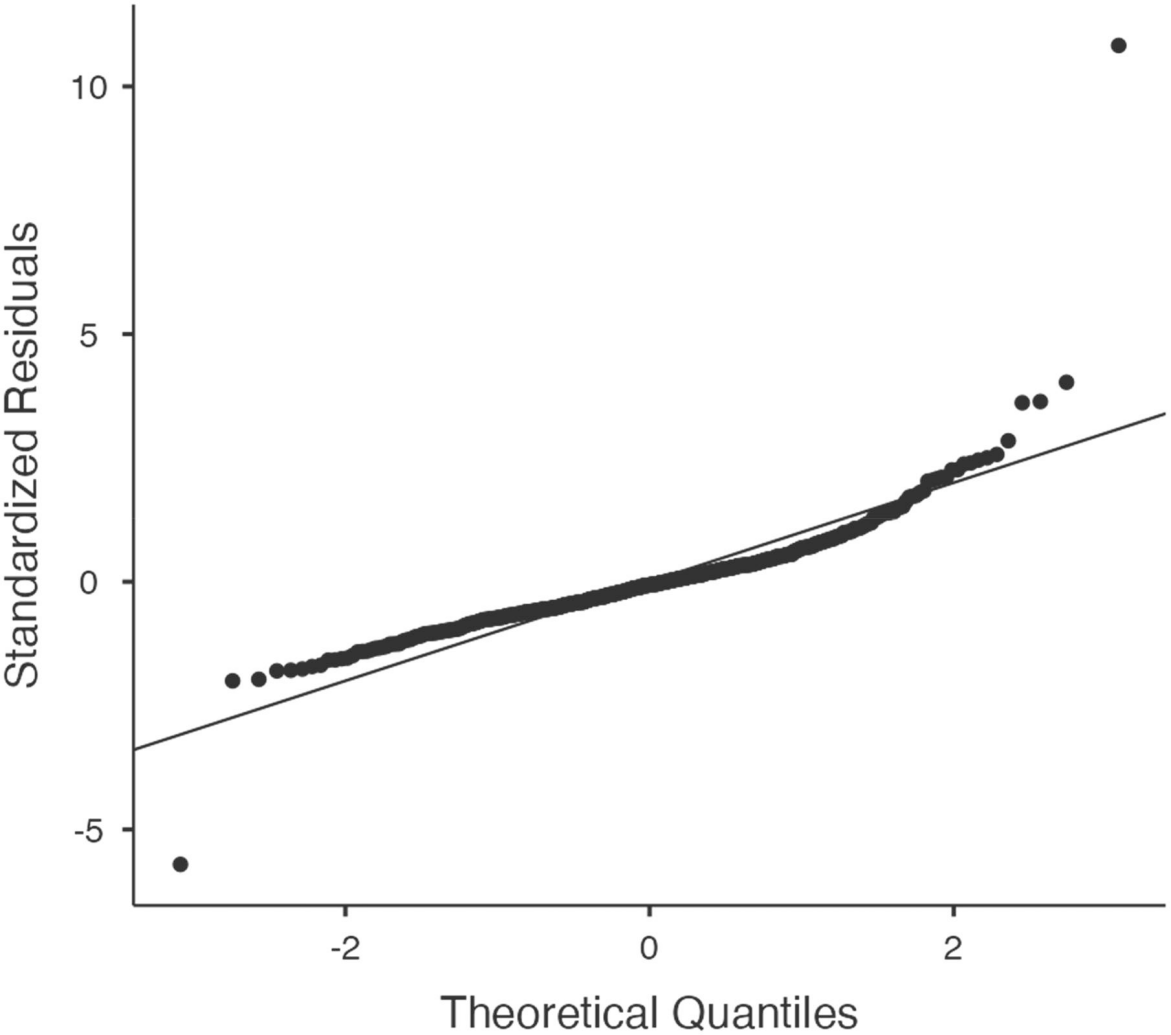
Normal Q–Q plot of residuals from the multiple linear regression model predicting actual body weight. Given the large sample size (*N* = 491) and the robustness implied by the central limit theorem, these minor deviations in the tails were deemed negligible for the overall stability of the regression estimates.

**Table 7:** Multiple linear regression model predicting actual body weight from mid-arm circumference, height, and habitus score, including multicollinearity and residual diagnostics.

Analysis/test group	Variable/test name	Coefficient/statistic	Standard error (SE)/tolerance	*t*/VIF/DW	*p*-value
Model coefficients	Intercept	−48.464	1.3714	−35.34 (*t*)	<0.001
	HS	2.932	0.4874	6.02 (*t*)	<0.001
	MAC	1.877	0.1460	12.85 (*t*)	<0.001
	Height	0.294	0.0175	16.77 (*t*)	<0.001
Collinearity statistics	HS	–	0.634 (Tolerance)	1.58 (VIF)	–
	MAC	–	0.193 (Tolerance)	5.17 (VIF)	–
	Height	–	0.228 (Tolerance)	4.38 (VIF)	–
Autocorrelation	Durbin–Watson	0.00743 (Autocorr.)	–	1.98 (DW)	0.816
Normality tests	Shapiro–Wilk	0.611	–	–	<0.001
	Kolmogorov–Smirnov	0.116	–	–	<0.001
	Anderson–Darling	11.90	–	–	<0.001
Heteroskedasticity	Breusch–Pagan	6.22	–	–	0.102
	Goldfeld–Quandt	2.08	–	–	<0.001
	Harrison–McCabe	0.326	–	–	<0.001

### Weight estimation accuracy

The performance and clinical accuracy of the two linear regression models across pediatric age categories were measured (see Table 8). In the initial linear regression model using only MAC measurement (model 1), the overall performance fell significantly below the recommended clinical benchmarks, yielding a total PW10 of 10.8% and a total PW20 of 21.0%. Stratified analysis by age cohorts revealed a clear, age-dependent upward trend in accuracy; however, unacceptable clinical performance was observed in the youngest cohorts. Specifically, the single-variable model virtually failed in the infant group (PW10: 2.0%, PW20: 2.0%) and the preschool group (PW10: 0.0%, PW20: 1.1%). Performance showed intermediate improvement in the school-age cohort (PW10: 10.1%, PW20: 22.2%), whereas the highest precision was achieved in the oldest subgroup, namely the adolescent cohort (PW10: 41.9%, PW20: 81.7%).

**Table 8:** Comparison of clinical accuracy between the single-variable model (model 1) and the multiple linear regression model (model 2) across pediatric age groups.

Age group	Count (*n*)	Model 1: only MAC	Model 2: MAC + height + HS
Infant (0–2 years)	205	PW10: 4 (2.0%) PW20: 4 (2.0%)	PW10: 38 (18.5%) PW20: 70 (34.1%)
Preschool (2–6 years)	94	PW10: 0 (0.0%) PW20: 1 (1.1%)	PW10: 25 (26.6%) PW20: 46 (48.9%)
School-age (7–12 years)	99	PW10: 10 (10.1%) PW20: 22 (22.2%)	PW10: 49 (49.5%) PW20: 79 (79.8%)
Adolescent (12 years or older)	93	PW10: 39 (41.9%) PW20: 76 (81.7%)	PW10: 63 (67.7%) PW20: 86 (92.5%)

MAC, mid-arm circumference; HS, habitus score; n/count, number of subjects.

Model 2 includes MAC, height, and HS as independent predictors.

Accuracy is presented as the number and percentage of children whose predicted weights fell within ±10% (PW10) or ±20% (PW20) of their actual measured weights.

In contrast, the newly developed multiple linear regression model incorporating MAC, height, and habitus score (model 2) yielded a substantially higher predictive power (R^2^ = 0.893, *p* < 0.001). With the inclusion of height and HS variables, the clinical accuracy improved remarkably across all pediatric age categories. Most notably, the PW20 accuracy in the vulnerable infant group increased from 2.0% to 34.1% (70/205), while the preschool group increased to 48.9% (46/94). Furthermore, the advanced model demonstrated the highest precision in the adolescent group, with an exceptional PW20 rate of 92.5% (86/93). These findings clearly demonstrate that while utilizing MAC as a standalone predictor exhibits highly variable, age-restricted performance, integrating height and habitus score effectively overcomes these limitations and provides a significantly more robust approach for pediatric weight estimation.

The initial linear regression model using only MAC measurement demonstrated highly age-dependent and variable performance, dropping to an unacceptably low PW20 accuracy of 2.0% in the infant group (0–2 years) (Table 8). In contrast, with the addition of the height and HS variables in the multiple linear regression model, clinical accuracy improved substantially across all age groups. Most notably, the PW20 accuracy in the infant group remarkably increased from 2.0% to 34.1% (70/205), while the preschool group reached 48.9% (46/94). Furthermore, the advanced model achieved its highest accuracy in the adolescent group (12 years or more) with an exceptional PW20 rate of 92.5% (86/93).

### Agreement analysis

Overall agreement in our study was 74.3%. A regression Bland–Altman plot (R^2^ = 0.878) indicated strong agreement (Figures 4, 5).

**Figure 4:**
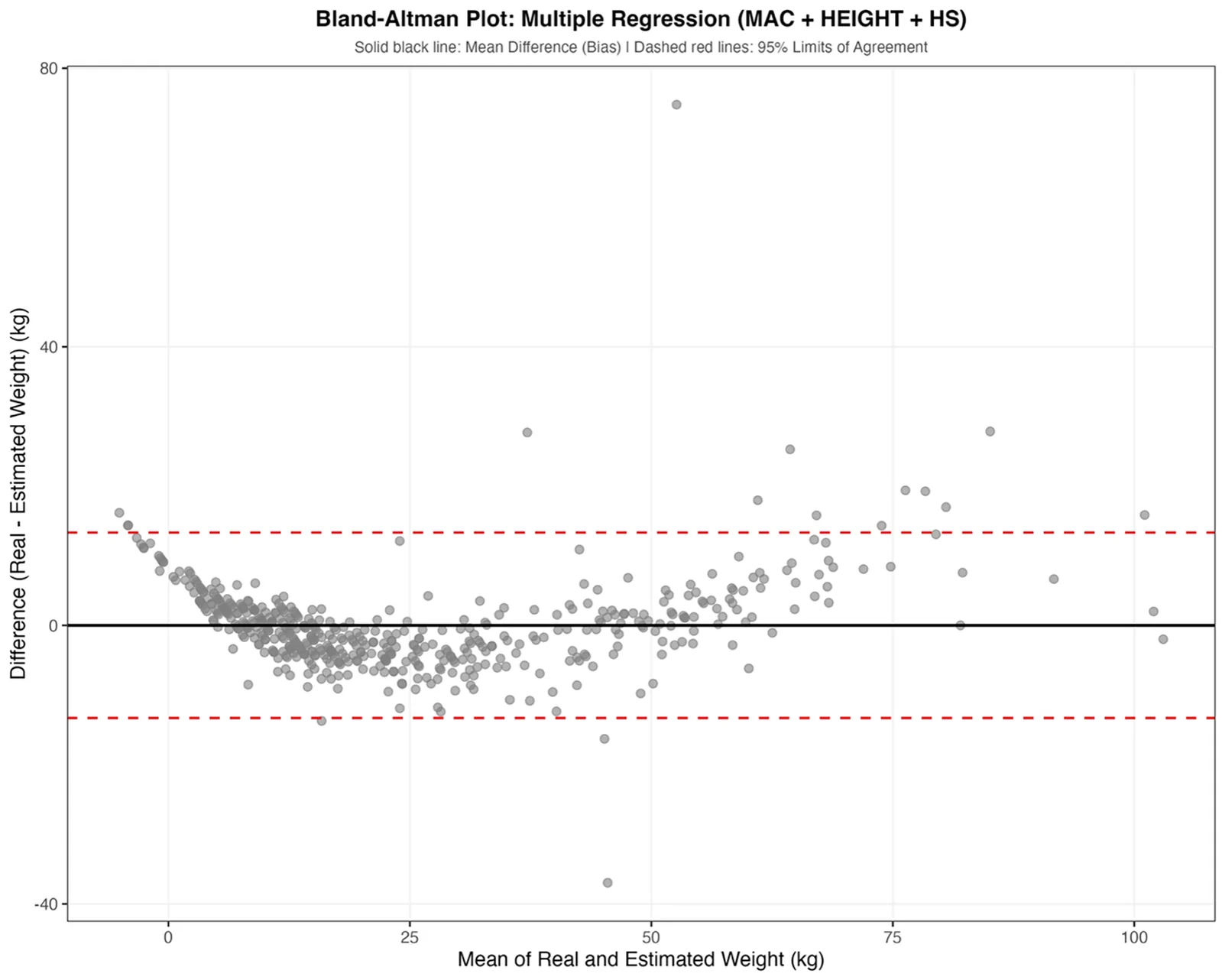
Bland–Altman plot assessing agreement between actual measured weight and weight predicted by the multiple linear regression model (MAC + height + habitus score).

**Figure 5:**
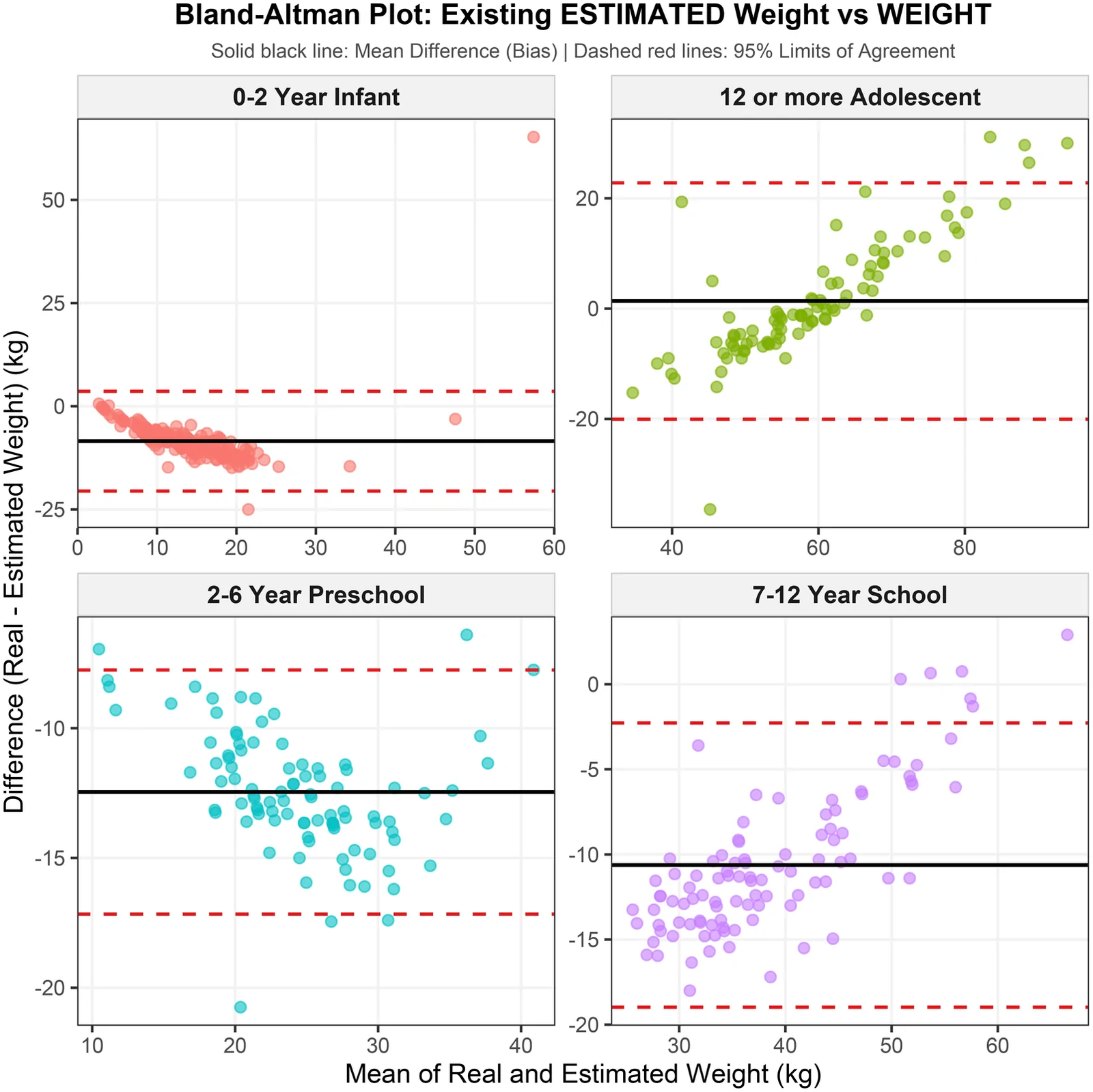
Age-stratified Bland–Altman plots showing agreement between actual and estimated weights across the infant, preschool, school-age, and adolescent groups.

Across the clinical weight spectrum, integrating height and habitus score effectively stabilized the variance of differences compared to the single-variable approach. While individual-level weight estimations inherently carry wider agreement limits due to the broad weight distribution across the pediatric sample, the multiple regression model effectively mitigated the substantial systematic error (bias) previously observed in the youngest cohort. In the infant cohort, the mean difference line shifted closer toward the zero line.

In contrast to the single-variable estimation approach, the multiple linear regression model slightly improved systematic bias across the distinct age cohorts. In the preschool and school-age cohorts, the previous persistent overestimation trends and wide downward scatters changed, with the majority of the residuals clustering more tightly around the zero baseline, indicating a substantial improvement in individual-level precision. Furthermore, in the adolescent cohort, the profound positive proportional bias and heteroscedasticity observed at the baseline scale were mitigated. Rather than exhibiting a rapidly increasing error rate or severe underestimation for heavier adolescent subjects near 100 kg, the advanced model maintained stable, controlled variance across the entire weight spectrum (40–100 kg). Although estimations differed across the age groups, with overestimation at younger ages and underestimation for adolescents, these improvements confirm that capturing both linear growth (height) and physical build (habitus score) prevents the prediction limits from expanding unacceptably, thereby ensuring clinical safety and reliable performance for pediatric subjects across all growth stages.

### Structural validity findings

All the investigated variables demonstrated robust factor loadings onto the primary underlying construct. Weight and MAC exhibited the strongest structural relationships, with factor loadings of 0.89 and 0.85, respectively. HS also showed a strong contribution, with a loading of 0.68, while weight ratio provided a moderate but statistically meaningful contribution at 0.52. These uniform high loadings confirm that all four variables are strong and valid indicators for estimating pediatric weight. In terms of factor loadings, the model generally explained 62.4% of the variance, and this value is acceptable for a single-factor scale and indicates strong construct validity. In particular, MAC's high factor loading confirms that the PAWPER XL-MAC scale is consistent in its aim of supporting subjective habitus scoring with objective anthropometric measurements. Consequently, this consistent structure among the variables provides the scale with structural validity in the Turkish pediatric population (Table 9).

**Table 9:** Factor loadings from the single-factor principal component analysis for habitus score, mid-arm circumference, body weight, and weight ratio.

Variable	Factor loading
Habitus score	0.68
Mid-arm circumference	0.85
Weight	0.89
Weight ratio	0.52

The single-factor PCA results showed that the HS, MAC, weight, and weight ratio variables strongly clustered under a single latent “body habitus” factor. CFA proxy metrics also indicated that the overall fit of the model was in the acceptable-to-good range, with CFI (∼0.92–0.95) and SRMR (∼0.06–0.08) values supporting the scale's structural validity. The RMSEA (∼0.085–0.11) remained within the acceptable/borderline range, indicating minor residual error. The TLI (∼0.88–0.93) was in the acceptable-to-good range, indicating adequate comparative fit relative to a null model. Chi2/df values (∼3.2–4.1) consistently fell below the acceptable threshold of 5, supporting a parsimonious model structure (Table 10).

**Table 10:** Confirmatory factor analysis proxy fit indices for the single-factor structural model of the PAWPER XL-MAC scale in Turkish children.

Metric	Value	Acceptable threshold	Comment
CFI	∼0.92–0.95	>0.90 (good > 0.95)	Good fit
TLI	∼0.88–0.93	>0.90	Acceptable – good
RMSEA	∼0.085–0.11	<0.08 (good <0.06)	Acceptable (borderline)
SRMR	∼0.06–0.08	<0.08	Good
*χ*^2^/df	∼3.2–4.1	<5 (preferably <3)	Acceptable

## Discussion

Child weight estimations vary by region, methods, and validations (4). The PAWPER XL-MAC scale was validated in Turkish children in this study involving 491 patients aged 0–18 years. The scale showed strong structural validity. A regression model with MAC, height, and HS had excellent explanatory power (R^2^ = 0.878), with acceptable multicollinearity and model assumptions. PCA showed HS, MAC, weight, and weight ratio clustered under a single “body habitus” factor, explaining 62.4% of the variance, with weight (0.89) and MAC (0.85) loading strongly. CFA metrics indicated a good model fit (CFI ∼0.92–0.95, SRMR ∼0.06–0.08), confirming the scale's validity in Turkish children.

The positive correlation between MAC and HS (*r* = 0.399, *p* < 0.001) and the increase in MAC and weight across HS categories confirm MAC's role in supporting subjective habitus assessment. This aligns with validation studies and highlights MAC's value in enhancing reliability, especially in populations with varied obesity prevalence (1). As Wells et al. noted, MAC significantly contributes to habitus evaluation and inter-rater consistency (1). With rising childhood obesity and socioeconomic influences in Turkey, objectifying habitus scoring via MAC offers a clinically important advantage.

Despite differing body composition and anthropometric trajectories between boys and girls during childhood and adolescence, the subgroup analyses in this study showed the PAWPER XL-MAC scale performed consistently across sexes. This matches previous studies reporting similar accuracy metrics in both genders and the finding that sex was not a major factor affecting the tool's performance (5). The findings suggest that while physiological HS are similar for boys and girls, indicators such as MAC and total body weight vary significantly by gender. This underscores the importance of considering gender differences when validating pediatric weight estimation tools.

The analysis by age showed distinct growth patterns. Age correlated strongly with weight (*r* = 0.875) and MAC (*r* = 0.808), fitting pediatric growth norms. The correlation with HS was weak (*r* = 0.107), indicating that somatotype remains stable across ages while physical dimensions change quickly. Weight estimation accuracy clearly depends on age. The PW10 (10.8%) and PW20 (21.0%) were below clinical benchmarks of PW10 > 70% and PW20 > 95% (1, 2, 6, 7, 9–12). Performance was lowest in children aged 0–2 years (PW10 2.0%, PW20 2.0%) and 2–6 years (PW10 0.0%, PW20 1.1%), improving with age. The highest measurement performance was observed in children between 0−2 and 5−12 years old. Nah et al. only reported children aged 6−12 years but found similar results (8). Some studies have indicated that weight estimation varies with age and habitus across populations (13). Wells et al. noted that interpreting results across age groups from the PAWPER XL-MAC scale is challenging (1).

Although HS is partially informed by height and MAC in the PAWPER XL-MAC framework, multicollinearity diagnostics confirmed that the predictors contributed sufficiently independent information to the model. The MAC + height + HS model outperformed the PAWPER XL-MAC, showing minimal bias but had proportional bias at weight extremes, especially in heavier adolescents. Bland–Altman plots confirmed overestimation at younger ages and underestimation in heavier adolescents, emphasizing the challenge of weight estimation in children and the need for age-specific adjustments.

The MAC-HS agreement, measured by Goodman–Kruskal’s gamma (*γ* = 0.226), showed a significant but moderate positive association, indicating MAC tracks habitus changes, but individual categorization may need visual assessment, especially in borderline cases. The weak-to-moderate strength of this association highlights that relying solely on upper arm thickness can introduce minor categorization discrepancies in an unselected pediatric population. Such variance is frequently encountered in critical care settings, where physiological confounders—including atypical skeletal builds, heterogeneous muscle-to-fat ratios, or localized tissue edema—can alter perimeter measurements without reflecting a true shift in the reference metabolic habitus. Consequently, our findings support the deployment of the PAWPER tool for rapid, objective weight triage, while reminding clinicians to synthesize solitary MAC assignments with qualitative visual assessments to achieve optimal dosing safety. In Nepal, better agreement was seen among those with HS < 3 on the PAWPER XL-MAC scale (10). Sivagni et al. found weight estimation was high at HS 5 and low at HS 1 in Italy (12). Some studies have reported that PAWPER XL-MAC underestimates obese children and overestimates underweight children (3). Despite this, it 's considered better than other scales (13). Our study had few obese or underweight children, so our conclusions are limited, but agreement was good for the underweight group.

High MAC-HS agreement rates were seen in the HS 1 (88.9%) and HS 3 groups (92.7%), but small patient numbers in the extreme habitus groups (HS 1: *n* = 9; HS 5–7: *n* = 10) limit these findings’ generalizability. Larger studies with more underweight and obese children are needed to better evaluate the PAWPER XL-MAC scale across all habitus scores.

In our study, we found significant increases in body weight, height, and MAC across higher HS groups (*p* < 0.001). This aligns with the PAWPER XL-MAC system, which uses arm circumference to assess body habitus and estimate pediatric weight. The *post-hoc* analysis showed that the higher HS groups (HS 4 and HS 5) had larger MAC and weight than the lower groups (HS 1, 2, and 3), demonstrating the tool's ability to distinguish thinner children from those with greater somatic development.

A minor constraint was the extreme scarcity of patients in the highest habitus tiers, HS 6 (*n* = 2) and HS 7 (*n* = 1), leading to their exclusion from inferential testing due to variance calculation issues. While this limits statistical generalization for the upper habitus spectrum, the robust differences among the other groups support the clinical utility of MAC-based stratification in pediatric emergency and critical care.

The strong correlations of age with weight (*r* = 0.875) and MAC (*r* = 0.808), versus the weak correlation with HS (*r* = 0.107), show habitus categories stay stable with age while measures increase with growth. This supports the use of the PAWPER XL-MAC scale and highlights the need to consider age and development in weight estimates.

For weight estimation scales, measurement suitability was evaluated using the PW10 > 70% and PW20 > 95% criteria (11). In the literature, the PAWPER XL-MAC scale has acceptable suitability, with PW10 > 70% and PW20 > 95% (1, 2, 6, 7, 9, 10, 12). Some studies have shown that PAWPER alone is superior to other estimation scales (14, 15). Studies have also indicated that the PAWPER XL-MAC performs better than other methods, such as Broselow (3, 9). In Ghana, it has been reported that even in children with chronic diseases such as sickle cell anemia, the PAWPER XL-MAC yielded results within an acceptable range of up to 20% of actual weight (9). In our study, the PW10 and PW20 values for MAC were consistent, especially in the 5–12 and 12+ age groups, while performance remained moderate or low in other age groups.

A key strength of the multiple linear regression model developed in this study is its ability to overcome these age-dependent performance limitations inherent in using MAC alone. While the single-variable MAC model failed dramatically in infants (0–2 years) with an unacceptably low PW20 accuracy of 2.0%, the integration of height and HS as independent predictors resolved this critical vulnerability, increasing the infant PW20 accuracy to 34.1%. This substantial improvement in the youngest cohort is clinically significant, as weight estimation in infants is notoriously challenging in emergency settings due to rapid body habitus changes. Furthermore, the advanced model achieved an exceptional PW20 accuracy of 92.5% in the adolescent group (12 years or older), demonstrating a robust predictive power (R^2^ = 0.893, *p* < 0.001) that is competitive with well-established international pediatric weight estimation systems. These findings underscore that while MAC provides a solid baseline for weight estimation, capturing structural diversity through height and physical build (habitus score) is mandatory to achieve acceptable overall clinical accuracy across all pediatric age categories, supporting the need for a comprehensive validation covering all habitus score and height categories in Turkish children.

The stratified Bland–Altman analysis provides critical insights into the clinical utility of the newly developed model by demonstrating how integrating height and HS directly rectifies the systematic prediction errors observed with standalone anthropometric measures. In pediatric resuscitation and emergency drug dosing, a major limitation of weight estimation tools is their tendency to yield proportional bias—either overestimating weight in younger cohorts, which carries a critical risk for medication overdosing, or severely underestimating heavier, obese adolescents (9, 11). Our advanced regression model successfully stabilized these variance disparities across the entire weight spectrum. The shifting of the mean difference line closer to zero in the infant group clinically validates that combining linear growth and physical build counters the age-dependent confounding effects of rapid adipose tissue changes. Furthermore, mitigating heteroscedasticity and preventing a rapidly increasing error rate in heavier adolescent subjects near 100 kg demonstrates that this approach maintains robust prediction limits. The Bland–Altman plots in this study showed that weight in young children was overestimated, and that weight was underestimated in adolescents. Therefore, the challenge of weight estimation in children is related to age.

In terms of the CFA proxy metrics, the model's fit is generally acceptable. The CFI (∼0.92−0.95) and TLI (∼0.88−0.93) values met the good fit threshold; the SRMR (∼0.06−0.08) shows near-perfect residual fit, while the RMSEA (∼0.085−0.11) and *χ*^2^/df (∼3.2−4.1) values remain in the borderline acceptable range. These proxy results suggest that the fit could be further improved if full CFA modeling was performed, because the high correlation between MAC and weight (0.712) already creates strong common variance. These findings obtained in a Turkish pediatric population support the construct validity of the PAWPER XL-MAC scale and present a validity profile consistent with the accuracy reports in the international literature.

## Limitations

This single-center study, conducted in a pediatric surgery outpatient clinic, may not fully represent the broader pediatric population, including healthy children or those presenting to emergency departments. The relatively small number of patients in the extreme habitus categories (particularly HS 6 and 7) limits a robust evaluation across the full spectrum of body types. Ethnic subgroup analyses were not performed, and the cross-sectional design precludes assessment of longitudinal changes.

## Conclusion

This prospective validation study demonstrates that the PAWPER XL-MAC scale has strong construct validity and acceptable criterion validity among Turkish children when MAC measurement is used. Although the overall accuracy fell below international benchmarks, particularly in younger age groups, the scale reliably reflected body habitus and showed promising performance in older children and adolescents. These findings support the clinical utility of the PAWPER XL-MAC in Turkish pediatric settings while highlighting the need for age-specific adjustments and multicenter studies with broader representation of extreme habitus categories. Routine integration of MAC-assisted habitus assessment has the potential to improve weight estimation accuracy and enhance the safety of medication dosing and resuscitation in pediatric emergency care.

## Data Availability

The raw data supporting the conclusions of this article will be made available by the authors, without undue reservation.
